# A Review on the Modification of Cellulose and Its Applications

**DOI:** 10.3390/polym14153206

**Published:** 2022-08-05

**Authors:** Tariq Aziz, Arshad Farid, Fazal Haq, Mehwish Kiran, Asmat Ullah, Kechun Zhang, Cheng Li, Shakira Ghazanfar, Hongyue Sun, Roh Ullah, Amjad Ali, Muhammad Muzammal, Muddaser Shah, Nosheen Akhtar, Samy Selim, Nashwa Hagagy, Mennatalla Samy, Soad K. Al Jaouni

**Affiliations:** 1 School of Engineering, Westlake University, Hangzhou 310024, China or; 2 Gomal Center of Biochemistry and Biotechnology, Gomal University, Dera Ismail Khan 29050, Pakistan; 3 Institute of Chemical Sciences, Gomal University, Dera Ismail Khan 29050, Pakistan; 4 Department of Horticulture, Faculty of Agriculture, Gomal University, Dera Ismail Khan 29050, Pakistan; 5 School of Pharmacy, Xi’an Jiaotong University, Xi’an 710021, China; 6 State Key Laboratory of Chemical Engineering, College of Chemical and Biological Engineering, Zhejiang University, Hangzhou 310027, China; 7 National Institute of Genomics and Advanced Biotechnology (NIGAB), National Agricultural Research Centre, Park Road, Islamabad 45500, Pakistan; 8 BW Advanced Materials Co. Ltd., Shanghai 200120, China; 9 School of Chemical and Biological Engineering, Beijing Institute of Technology (BIT), Beijing 100000, China; 10 Institute of Polymer Material, School of Material Science & Engineering, Jiangsu University, Zhenjiang 212013, China; 11 Department of Botany, Abdul Wali Khan University Mardan, Mardan 23200, Pakistan; 12 Natural and Medical Sciences Research Center, University of Nizwa, P.O. Box 33, Birkat Al Mauz, Nizwa 616, Oman; 13 Department of Biological Sciences, National University of Medical Sciences, Islamabad 44000, Pakistan; 14 Department of Clinical Laboratory Sciences, College of Applied Medical Sciences, Jouf University, Sakaka 72341, Saudi Arabia; 15 Biology Department, Faculty of Science & Arts, University of Jeddah, Khulais 21921, Saudi Arabia; 16 Botany Department, Faculty of Science, Suez Canal University, Ismailia 41522, Egypt; 17 Department of Communications and Computers Engineering, The Higher Institute of Engineering, El-Shorouk City 11837, Egypt; 18 Department of Hematology/Oncology, Abdu Latif Jameel Scientific Chair of Prophetic Medicine Application, Faculty of Medicine, King Abdulaziz University, Jeddah 21589, Saudi Arabia

**Keywords:** cellulose, biomass, green chemistry, sustainability, circular economy

## Abstract

The latest advancements in cellulose and its derivatives are the subject of this study. We summarize the characteristics, modifications, applications, and properties of cellulose. Here, we discuss new breakthroughs in modified cellulose that allow for enhanced control. In addition to standard approaches, improvements in different techniques employed for cellulose and its derivatives are the subject of this review. The various strategies for synthetic polymers are also discussed. The recent advancements in polymer production allow for more precise control, and make it possible to make functional celluloses with better physical qualities. For sustainability and environmental preservation, the development of cellulose green processing is the most abundant renewable substance in nature. The discovery of cellulose disintegration opens up new possibilities for sustainable techniques. Based on the review of recent scientific literature, we believe that additional chemical units of cellulose solubility should be used. This evaluation will evaluate the sustainability of biomass and processing the greenness for the long term. It appears not only crucial to dissolution, but also to the greenness of any process.

## 1. Introduction

Industries and customers are increasingly seeking biodegradable, [1,2] non-petroleum-based, [3] carbon-neutral products with environmental and safety hazards [4,5] derived from renewable and sustainable resources. Cellulose-based natural materials have been used in engineering in our society for millennia [6]. Their use continues to be seen in forest products, paper, textiles, packaging materials, and other industries [7]. These first-generation cellulose applications have an advantage in the hierarchical structure design. By utilizing a hierarchical structural design that spans nanoscale to macroscopic dimensions, natural materials produce high mechanical strength and flexibility performance [8,9].

On the other hand, traditional cellulosic materials cannot provide the characteristics and functionality for engineering applications. These types of forest products have their place, but they cannot match the high-performance material demands of modern society [10,11]. Sustainability necessitates the advancement of human science and technology, as well as a greater demand for trees, [12,13,14] plants, some marine organisms, and algae having a fundamental reinforcing property that enhances all subsequent constructions. The bulk of the hierarchical structure is eliminated by extracting cellulose at the nanoscale, and a new cellulose-based “building block” is available as composites [15,16].

**Figure 1:**
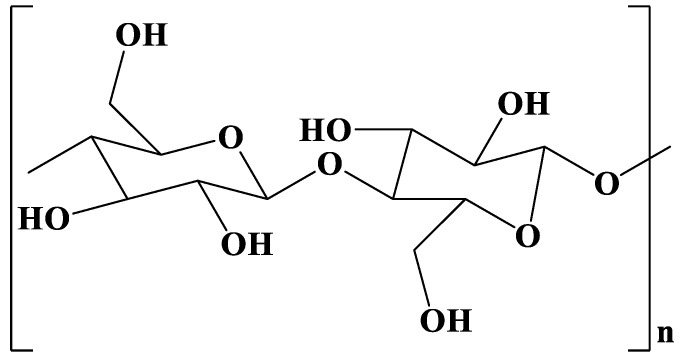
Chemical structure of cellulose.

Cellulose is made up of repeating anhydrous glucose units (AGU) that are covalently connected by acetal functionalities containing repeating units of cellulose OH groups. The main OH group along macromolecule chains is easily modified by interacting with functional groups. It results a wide range of cellulose derivatives [17]. AGU have a wide range of reactivity, which is influenced by the steric effects of reagents, and supramolecular structure [18]. Esterification, acylation, grafting, and etherification have all been used to make cellulose derivatives in solvent systems, as shown in Figure 1. Cellulose is an excellent candidate for the fabrication of sustainable materials, owing to its good availability, renewability, and biodegradability [19,20]. The efficient chemical modification of cellulose by its grafting commonly involves aprotic solvents, toxic reactants, and harsh reactant conditions, which have a negative effect, reduce the dispersibility, and require further purification.

### 1.1. Cellulose Source

Cellulose is extracted from raw biomass processing. There is a growing desire for new technologies to be developed. To overcome the disadvantages of cellulose, it is best to dissolve it. Processes for pulping and processing have been around for a long time. In this review, the authors look at the process of cellulose and its interactions. To cover every step of the subject that has been published, we give our thoughts on several aspects of cellulose processing. Greenness and sustainability are emphasized whenever possible. The fact must be acknowledged that, as new information becomes available, it must be evaluated on a regular basis [21,22,23].

### 1.2. Chemical Structure of Cellulose

Cellulose is a structural component of a green plant cell walls, also produced by algae, acetobacter, and rhizobium, among other organisms. All plant matter has a cellulose concentration of roughly 33%, on average. It is possible to extract cellulose from its raw biomass materials, and it has the potential to be a virtually limitless source of biofuel that is renewable. More emphasis is being placed on the use of such a biopolymer in the development of synthetic products that are environment friendly and biocompatible [24]. Cellulose is an organic compound with the formula a polysaccharide consisting of a linear chain of several hundred to many thousands of β (1→4) linked D glucose units. Cellulose is an important structural component of the primary cell wall of green plants, many forms of algae, and oomycetes. Some species of bacteria secrete it to form biofilms. Cellulose is the most abundant organic polymer on Earth. The cellulose content of cotton fiber is 90%, that of wood is 40–50%, and that of dried hemp is approximately 57%.

### 1.3. Advantages of Cellulose Materials

Due to its excellent performance, cellulose is a renewable and biodegradable natural polymer. Moreover, it has other advantages, such as low density, high porosity, and a large specific surface area. Thus, it can be applied for many purposes in the areas of adsorption and oil/water separation, thermal insulation, and biomedical applications, as well as many other fields [25,26]. Natural cellulose, mainly obtained from bacterial (BC) and plant-based (PC) sources, can serve as a high-potential scaffold material for different regenerative purposes. Natural cellulose has drawn the attention of researchers due to its advantages over synthetic cellulose, including its availability, cost-effectiveness, per usability, biocompatibility, negligible toxicity, mild immune response, and imitation of native tissues.

### 1.4. Limitations of Traditional Cellulose Materials

Over the last few decades, there has been a lot of research on cellulose-based particles and composites. The various aspects of cellulose processing, their chemical modification, rheological behavior, suspensions, and water interaction were discussed with an explanation [27]. Furthermore, modeling of the crystalline structure, as well as the use of analytical models, gives a method for evaluating the potential of cellulose composites [28].

### 1.5. Advantages of CNC to Overcome the Limitations

Cellulose nanoparticles (CNCs) are appropriate materials for a new biopolymer composites industry to be built on. The axial elastic modulus of crystalline cellulose is higher than that of Kevlar, as comparable to other reinforcement materials [29]. CNCs have a low density, and a reactive surface with OH side groups, which allows chemical species to be grafted on its surface. Their surface functionalization enables customizing to aid self-assembly, and controlled dispersion in a matrix polymer for binding strength [30]. A transparent solution has shown tensile strengths greater than cast iron using a range of different CNCs composites [31,32]. This type of CNC is used in barrier films, flexible displays, and reinforcing fillers for polymers in biomedical [33] pharmaceuticals and drug delivery, templates for electronic components, and many other applications [34].

### 1.6. Recent Advantages on Cellulose Research

In the current global context, cellulose fulfills the characteristics that give it clear advantages over synthetic fibers, having a huge potential for substituting fossil-based materials which are polluting and are harmful to ecosystems [35]. Research conducted in most laboratories around the world in the field of cellulose is overwhelmingly aimed at industrial needs because features such as renewability and low cost are the most important attributes for economic success. Cellulose continues to display its advantages over synthetic fibers and its potential to replace fossil-based materials, which are known to harm ecosystems. Common attractive applications of cellulose include packaging, healthcare materials, electronics, and printing. Most applications seem to rotate around the equilibrium of hydrophilicity, its mechanical properties, and optical properties. Details on industrial applications, knowledge gaps, and green innovations in cellulose conductivity, as well as limitations of its thermal degradation, are thoroughly covered. Most innovations are motivated by industrial needs because renewability and inexpensiveness are the latest additional values to most industries [36,37].

### 1.7. Cellulose Extraction

Based on the formic acid process, cellulose was extracted from different plant fibers, with a 59.8% yield. The fibers have 10.9% lignin content. The acid hydrolysate of cellulose contained 2.7% glucose and 0.2% xylose. It was demonstrated that 76% of hemicelluloses and 85.8% of lignin in jute could be extracted. Moreover, formic acid (20%) and hydrogen peroxide (10%) were used in the extraction of cellulose (60%) with α-cellulose (93.7%), with 70% crystallinity from oil palm empty fruit bunches (OPEFB). The commercially available microcrystalline cellulose (MCC), [38] and the alkali and bleaching treatments on fibers extracted from oil palm fronds succeeded in extracting cellulose, with 40% yield on a dry weight basis. Cellulose fibers and cellulose nanocrystals were extracted from rice husks [39,40]. Fibers were obtained by submitting the industrial rice crop to alkali (NaOH) and bleaching treatments. Nanocrystals were extracted from these fibers using sulfuric acid (H_2_SO_4_) hydrolysis treatment [41,42].

In this review, the various strategies for synthetic polymers are also discussed. The recent advancements in polymer production allow for more precise control, and make it possible to make functional celluloses with better physical qualities. For sustainability and environmental preservation, the development of cellulose green processing is the most abundant renewable substance in nature. The discovery of cellulose disintegration opens up new possibilities for sustainable techniques. Based on the review of recent scientific literature, we believe that additional chemical units of cellulose solubility should be used. This evaluation will evaluate the sustainability of biomass and processing the greenness for the long term. It appears the choices are not only crucial to dissolution, but also to the greenness of any process.

## 2. Different Techniques of Cellulose Modification

**Figure 2:**
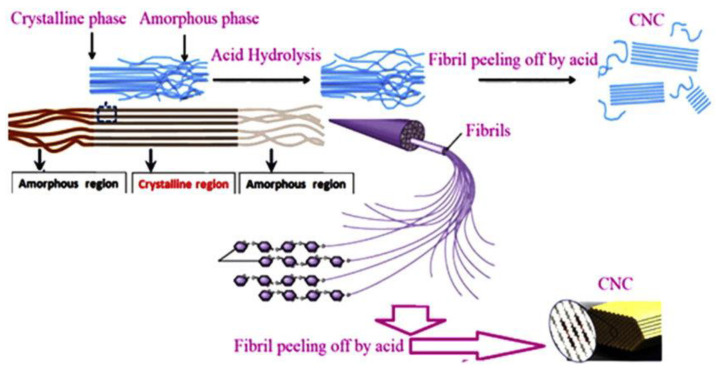
Extraction of cellulose nanocrystals. (Copyright and permission, Elsevier—2022).

Despite the fact that the bioplastic industry is growing rapidly with many innovative discoveries, cellulose-based bioproducts in their natural state face challenges in replacing synthetic plastics. These challenges include scalability issues, a high cost of production, and, most importantly, the limited functionality of cellulosic materials. However, in order for cellulosic materials to be able to compete with synthetic plastics, they must possess properties adequate for the end user, and meet performance expectations. In this regard, the surface modification of pre-made cellulosic materials preserves the chemical profile of cellulose, its mechanical properties, and biodegradability, while diversifying its possible applications. Cellulose has been subjected to a wide range of chemical modifications towards increasing its potential in certain fields of interest, as shown in Figure 2 [43].

These modifications either modulated the chemistry or introduced certain functional groups onto its surface, which varied from simple molecules to polymers. Among many, aliphatic and aromatic mono- and di-isocyanates are a group of chemicals that have been used for a century to modify cellulose.

### 2.1. Cellulose Surface Modification

The cellulose surface modification significantly increased its potential due to its OH group, whereas on the other side, it is difficult to control the reaction between the di-isocyanate and cellulose [44]. There are various types of surface modification described with detail [45].

### 2.2. Reaction with Carboxylic Acid Groups

The abundance of carboxylic acid groups on TEMPO-oxidized (2,2,6,6-tetramethylp peridine 1-oxyl radical) reactions has been extensively studied to functionalize the surfaces. The reaction with Lissamine rhodamine B ethylenediamine is frequently used to attach fluorophores to TEMPO-oxidized cellulose. It is essential to synthesize activated esters as intermediates, such as N-hydroxysuccinimide. The tetrazole-based nitrile imine carboxylic acid ligation method can be used directly to functionalize TEMPO-oxidized cellulose [46,47,48]. The fluorescent creation of benzohydrazide was utilized for monitoring the interaction with the cell’s advantage. Multicomponent reactions, involving, for example, carboxylic acid for the Passerini reaction, show a possible efficiency. In addition, cellulose is separated by H_2_SO_4_ treatment with an azetidinium salt. Pre-modification is required for indirect surface modification. The increased activity of the functional group is commonly the case for the additional functionality. It could be a one- or two-step process. Two-step procedures allow for the introduction of a variety of functional groups, including various dyes. Carboxylate functions may not be present depending on the cellulose isolation process. In that instance, amine groups can be added to a coupling reaction between the amines’ activated esters. This method has been used to adhere the fluorescent dye to cellulose on numerous occasions [49]. This type of amine was added by using epoxide chemistry, followed by isothiocyanate reactions. Without the introduction of any strong mineral acids, a high yield of up to 60% of pure carboxylic cellulose has been successfully produced, but the hydroxy and carboxylic acid group treated with water molecules adsorbed in a specific way, which varies with the type of polar group.

### 2.3. Alkyne–Azide Reaction

The insertion of an alkyne functionality reacts with the surface of cellulose. At room temperature, 1-azido-2,3-epoxypropane reacts with the OH groups of cellulose. To achieve this, the propargylic groups, propargylamine and propargyl-modified 4,6-dichloro-1,3,5-triazine, can also be created. The effective copper-catalyzed reaction allows a variety of groups, such as gold nanoparticles (AuNPs) carrying dendrimers and b-cyclodextrin. The common reaction of cellulose with poly(e-caprolactone) PCL and poly(ethyl ethylene phosphate) PEEP was investigated [50,51].

### 2.4. Diels–Alder Cycloaddition

**Figure 3:**
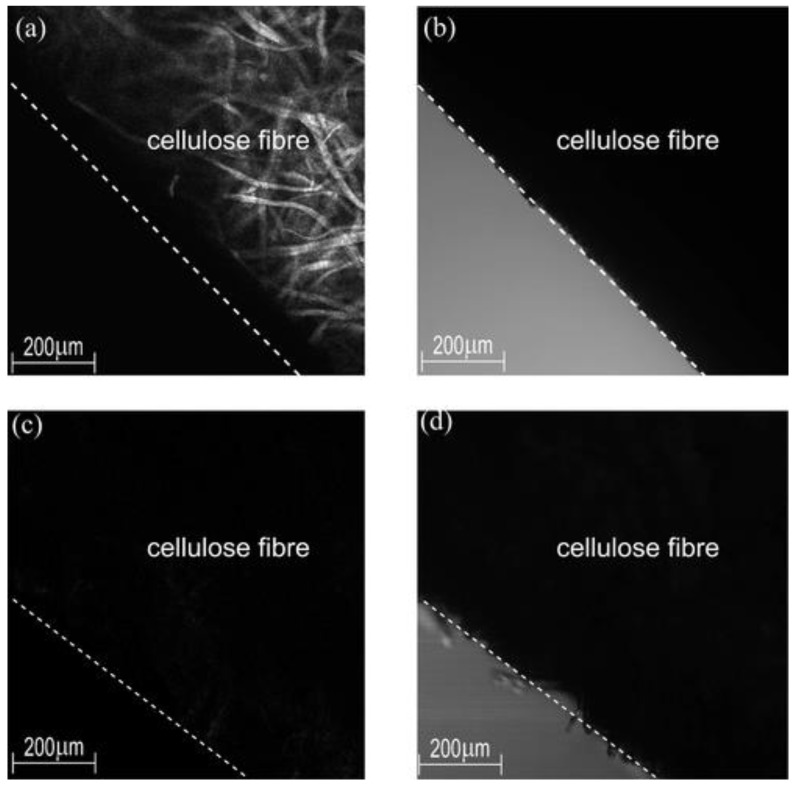
Fluorescence spectroscopy images. (**a**) Cel-*g*-*N*-(1-pyrene)-maleimide, (**b**) Cel-*g*-*N*-(1-pyrene)-maleimide, (**c**) blank sample, and (**d**) transmission image of blank sample. (Copyright and permission, ACS publication 2022).

Diels–Alder (DA) reactions are appealing because of their efficiency in the presence of other functional groups. Cellulose with a maleimide moiety was easily modified with functional molecules. Several colors, as shown in Figure 3, were conjugated to the tagged cellulose, allowing it to be monitored in a biological context. A combination of reversible addition-fragmentation chain transfer (RAFT) polymerization and hetero Diels-Alder (HDA) cycloaddition was used under mild, fast, and modular conditions. Poly(isobornyl acrylate) was grafted onto a solid cellulose substrate [52]. The active hydroxyl groups expressed on the cellulose fibers were converted to tosylate leaving groups, which were subsequently substituted by a highly reactive cyclopentadienyl functionality (Cp). By employing the reactive Cp-functionality as a diene, thiocarbonyl thio-capped poly(isobornyl acrylate), synthesized via RAFT polymerization (mediated by benzyl pyridine-2-yldithioformiate (BPDF), was attached to the surface under ambient conditions by an HDA cycloaddition. The analytical results provide strong evidence that the reaction of suitable dienophiles with Cp-functional cellulose proceeds under mild reaction conditions in an efficient fashion. In particular, the visualization of individual modified cellulose fibers has a homogeneous distribution of the polymer film on the cellulose fibers. Well-defined cellulose-graft-polyacrylamide copolymers were synthesized in a grafting-from approach by reversible addition-fragmentation chain transfer polymerization (RAFT). A chlorine moiety was introduced into the cellulose using 1-butyl-3-methylimidazolium chloride (BMIMCl) as solvent, before being substituted by a trithiocarbonate moiety, resulting in cellulose macro-chain transfer agents (cellulose-CTA) with DS(RAFT) of 0.26 and 0.41. Poly(N,N-diethylacrylamide) (PDEAAm) and poly(N-isopropylacrylamide) (PNIPAM) were subsequently grafted from these cellulose-CTAs and the polymerization kinetics [53,54,55]. The behavior of the cellulose-graft-copolymers was studied via the determination of cloud point temperatures, evidencing that the thermos responsive properties of the hybrid materials could be finely tuned between 18 and 26 °C for PDEAAm, and between 22 and 26 °C for PNIPAM side chains.

### 2.5. Photo-Thiol-Ene Reaction

These reactions containing radical have primarily been utilized to create surface-modified cellulose. They have recently been investigated as a means of producing water-cellulose-based drug carriers. The tetrazole-ene cycloaddition process is mediated by a light-driven nitrile imine. This type of reaction is a promising light-induced ligation. It is used for non-activated alkenes as a partner. The fluorescence of the resultant pyrazoline cycloadducts alkenes [56] showed varying emissions, such as 487–538 nm, depending on their structure. It is one of the most appealing aspects of this type of reaction. This enables the creation of cellulose with built-in fluorescence, [57] allowing for direct material monitoring in a biological setting [58]. These characteristics have not yet been investigated in the production of water-soluble cellulose. They have been examined in the production of fluorescent hydrogels containing cellulose [59].

### 2.6. Isotropically Modified Cellulose

Grafting on terminal groups which affect the end functionality can react with amines in a ring-opening process [60]. Carboxylic acid and polysaccharides such as cellulose can be selectively changed solely at the end group. This method allows the cylinder-shaped cellulose to be bound to a surface solely by the base, because the cellulose functional end can be attached to numerous scaffolds. This opened the door to several unique architectures. The oxidation of the reducing end functionality is followed by amidation to attach biotin, which contains binding sites, resulting in cellulose with four arms [61]. These types of modification exhibit excellent transparency, super flexibility, and outstanding mechanical strength and electric insulation, making them very promising for the highly efficient heat dissipation of diverse electronic devices. Their limited thermal conductivity seriously hinders their practical application in high-heat generation devices.

### 2.7. Cellulose in Ionic Liquids by Esterification and Acylation

Currently, esterified cellulose has gained the greatest attention for researchers. In general, cellulose conversion can be accomplished in the presence of catalysts by different routes [62]. The esterification with acylation, acyl chlorides, and anhydrides is used for the manufacture of esters, including aliphatic and aromatic [63].

Cellulose can be acetylated with different type of reagents, e.g., acetyl chloride, acetic anhydride, and vinyl acetate. These solvents have good cellulose solubilization properties, and can even act as organocatalysts [64]. Kakuchi et al. used the [EMIM][OAc] as a dual function (dissolving capability and organocatalytic property) for cellulose acetylation, obtaining a highly substituted cellulose triacetate reaction with IpeAc media proceeding smoothly and quickly [65]. A superbase-derived [DBNH][OAc] was used to acetylate cellulose efficiently with acetic anhydride. The reactions were carried out without the use of catalysts at mild temperatures (60–70 °C for 1 h). It was discovered that acetylation resulted in cellulose acetate (CA) with a degree of substitution (DS) of 2.97.

Researchers have used binary mixes and co-solvents as acylation mediums. DMSO, DMAc, ACN, and acetone are common co-solvents that are thought to aid in the acetylation process [66,67]. In [EMIM][OAc] with a co-solvent of DMSO, a gram-scale manufacture of polysaccharide acetates was obtained. The nucleophilic support is provided by anions of alcoholic species. For the organocatalytic capacity, cosolvent worked as an efficient accelerator. The synthesis of CA in binary solvents of [DBNH][OAc] shows dispersants. Using five anhydrous glucose units (AGU) at 80 °C, DS of CA reached 2.68 within 0.5 h. Similar conditions, without the addition of a co-solvent, resulted in a DS of 0.9 after 3.0 h. The protic with amidine and acetic acid for cellulose dissolving found that [DBUH][OAc] had the greatest catalytic performance for cellulose acetylation [68,69]. The DS of CA increased from 2.61 to 2.87. An ammonium base was used with acetone. The greatest DS value of the synthesized CA was 2.79.

Cellulose esters with various aliphatic groups are made of different types of reactants. Vinyl esters are commonly used as acylating agents in the manufacture of aliphatic cellulose esters. This type of cellulose modification was carried out in [EMIM][OAc] without external catalysts. As a result, aliphatic groups with chain lengths were introduced to the cellulose backbone [70]. Vinyl-laurate-modified cellulose with a DS of 2.4 was achieved at 80 °C with molar ratio of 3 AGU for 4 h, whereas vinyl laurate with a DS of 2.65 to 2.73 was synthesized at 120 °C for 10 min. A combination of [EMIM][OAc] and Valero lactone of homogenous cellulose laurate and cellulose chloroacetate were produced with different degrees of substitution. To make esterified cellulose, researchers use relatively inactive, readily available alkyl esters because alkyl esters produce esters with lower DS values. As for the acylation of cellulose, both mixtures have hydrophilic [BMIM][Cl] and a hydrophobic [BMIM][BF4]. In the presence of a lipase catalyst, cellulose was acylated with methyl esters. Long-chain cellulose esters with DS ranged from 0.213 to 1.452. 1-ethyl-3-methylimidazoium 2,4-dimethoxybenzoate acylation with methyl laurate was attempted ([EMIM][DMBz]) [71].

The reaction happened without the use of external catalysts with the help of DMSO as a co-solvent, yielding cellulose laurate with a DS of 0.52. Cellulose provides aliphatic esters with a pendant carboxylic acid group, making a ring-opening esterification of cyclic anhydrides, e.g., succinic anhydride (SA), maleic anhydride (MA). In this regard, reaction media, [AMIM][Cl] and [BMIM][Cl], are commonly used. By modifying AMIMCl/DMSO combinations with succinic anhydride, succinylated cellulose with a DS of 0.37 was generated. Cyclic anhydrides were used as reagents to alter cellulose catalyst, N-bromosuccinimide, to perform a succinylation reaction in a binary solvent of [BMIM][Cl]/DMSO (NBS). Cellulose, hemicellulose, and lignin were separated from bagasse and homogeneously esterified with GA in [AMIM][Cl] [72].

The reaction was extended to homogenous maleylation [73]. The percentage of substitution in maleylated cellulose was 13.5 percent. When it comes to cellulose aromatic esters, benzoyl chlorides with various p-substituted groups are commonly used to benzoylate the cellulose. In order to perform the reaction, a binary combination of 1-butylpyridinium chloride ([BPy][Cl]) and DMSO was used [74].

The reaction with p-iodobenzoyl chloride in [BMIM][Cl] yielded kraft cellulose p-iodobenzoate with DS values of 0.18–2.05. In N-methyl-N-(2-methoxyethyl) pyrrolidinium acetate ([P1ME][OAc]), homogeneous cellulose acylation with 2,2,2-trifluoroethyl benzoates resulted in cellulose benzoates with substitution (DS = 3). The reactions with 4-[(4-hydroxyphenyl)diazenyl]benzoate yielded cellulose benzoates with DS values of 2.38 and 2.67 in the same medium. The 3,5-dimethylphenyl isocyanate was used to react the unreacted OH groups at the 2 and 3-positions of the cellulose monoesters.

Cellulose benzoate propionates were made by adding propionic anhydride (Pr_2_O) and benzoic anhydride (Bz_2_O) in a tributylmethylammonium dimethyl phosphate (TBMADMP) in a stepwise manner. The benzoate was found in the C2 and C3 locations only. The completely substituted cellulose furoate and cellulose phenyl carbonate were accessible in the presence of pyridine. The phenolic acids were used to alter cellulose fibers, i.e., p-hydroxybenzoic acid, and vanillic acid. A recyclable mixture of tetrabutylammonium acetate and DMSO was used for homogenous esterification. Despite the fact that the DS of the cellulose derivative was less than 0.25, the generated films had good hydrophobicity characteristics [75].

### 2.8. Cellulose Modification by Etherification

The production of cellulose ethers, due to their initial basicity alkali aqueous systems, are ideally suited as homogenous reaction media [76]. In alkali aqueous systems, cellulose ethers such as methyl cellulose (MC), carboxymethyl cellulose (CMC), hydroxyethyl cellulose (HEC), hydroxy propyl methyl cellulose (HPC), and carboxyethyl cellulose (CEC) have been produced so far. Basically, these derivatives are made by reacting with etherifying agents, such as epoxides and alkyl halides [77].

**Figure 4:**
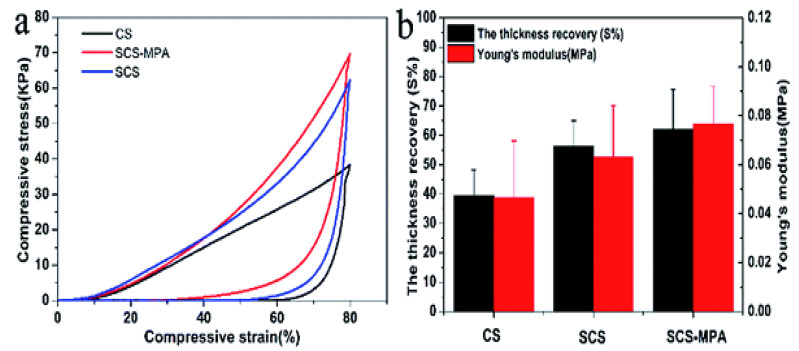
(**a**) Compressive stress–strain curves of unmodified (**b**) thickness recovery and Young’s modulus of unmodified silylated cellulose (CS), silylated cellulose sponge (SCS), and silylated cellulose sponge Young’s modulus (SCS-MPA). (Copyright and permission, RSC-2022).

Silylated cellulose sponge (SCS) can be subsequently functionalized with various thiol-containing compounds, such as 3-mercaptopropionic acid, via temperature-induced thiolene. The hydrophilic cellulose sponge displayed an excellent mechanical strength of 70 KPa at 80% compression strain. The prepared sponge features hydrophilic and underwater oleophobic properties, and was used in gravity-driven removals of oil-in-water emulsions, with a high separation efficiency, as shown in Figure 4 [78].

With n-dodecyl mercaptan (NDM), 2-aminoethanethiol hydrochloride (AET), and monothioglycerol (MG), amphiphilic cellulose (MCC-C16) improves the antifouling capabilities of the poly(acrylonitrile-co-methyl acrylate) ultrafiltration membrane. The reaction of MCC with 1-bromohexadecane in the mentioned aqueous solution (6 wt% NaOH/4 wt%) yielded MCC-C16. A solution of 7 wt% NaOH/12 wt% urea was used as a starting solvent for the cellulose etherification. Cellulose was dissolved in a 7 wt% NaOH/12 wt% urea solution without a catalyst, and was allowed to react with benzyl chloride (BC) under moderate circumstances [79].

Depending on the temperature and the molar ratio of BC, the benzyl cellulose showed DS values ranging from 0.29 to 0.54, respectively. The high-water concentration limits cellulose benzylation. It is difficult to get DS of pure cellulose than 7% NaOH/12 percent urea. The positive charge quaternary ammonium salt-modified cellulose (QMCC) was synthesized. The QMCC-added alkaline solid polyelectrolyte membrane enhanced the conductivity and tensile strength. It could be used in flexible Zn-air batteries. Cellulose with allyl glycidyl ether (AGE) in the same solvent produce 3-allyloxy-2- hydroxypropyl cellulose (AHP-celluloses), with DS ranging from 0.32–0.67, respectively.

Alkali cellulose also provides an ideal environment for the creation of cellulose-based hydrogels [80] due to the high water content and good dissolving ability. The chemical cross-linking of cellulose derivatives is used to make these hydrogels. Using 1,4-butanediol diglycidyl ether (BDE) in a 6 wt% NaOH/4 wt% urea solution yielded cellulose hydrogels [81]. To make cellulose ionic hydrogels with high elongation, [82] the cellulose is etherified with AGE to give it double bonds via chemical cross-linking.

On the other hand, vinyl compounds activated in such aqueous systems undergo a Michael addition reaction without a catalyst. The relative reactivity of OH groups in this situation was in the order C-6 > C-2 > C-3. In NaOH/urea solutions, cellulose polyelectrolytes with acylamino and carboxyl groups were produced uniformly. The Michael addition reaction with acrylamide was followed by the saponification of acylamino groups into carboxyl groups.

A cellulose amphiphilic derivative with hydrophobic benzyl and hydrophilic carboxyethyl groups was produced by etherifying with acrylamide and benzyl chloride. This type of reaction was carried out without the use of any additional catalysts in a NaOH/urea solution. Microcapsules are produced by cross-linking with polyurea. It is used for the encapsulation and controlled release of hydrophobic methyl 1-naphthylacetate. It also responsible for maintaining the pH and surfactant characteristics. For the cross-linking of cellulose in LiOH/urea solution, methylenebisacrylamide (MBA) was chosen as a Michael addition reagent [83]. A strong hydrogel was created, with a high water uptake capacity [84,85]. The optimal highly cationic charges, good stability, and acceptable thermostability might be considered as some of the alternative renewable reinforcement additives for nanocomposite production. The cellulose crystalline structure had a lower crystallinity than the starting cellulose.

### 2.9. Carbanilation

The carbanilation of cellulose can be performed by utilizing isocyanate as a reagent. In general [BMIM][Cl], Heinze’s group demonstrated polymerization of carbanilation with cellulose with three different degrees. In the absence of catalysts, the reaction was carried out with phenyl isocyanate. At 80 °C for 4 h, fully substituted cellulose carbanilates were generated using 10 anhydrous glucose units. The carbanilation was transferred to bacterial cellulose, which is very different from plant cellulose [86].

**Scheme 1:**
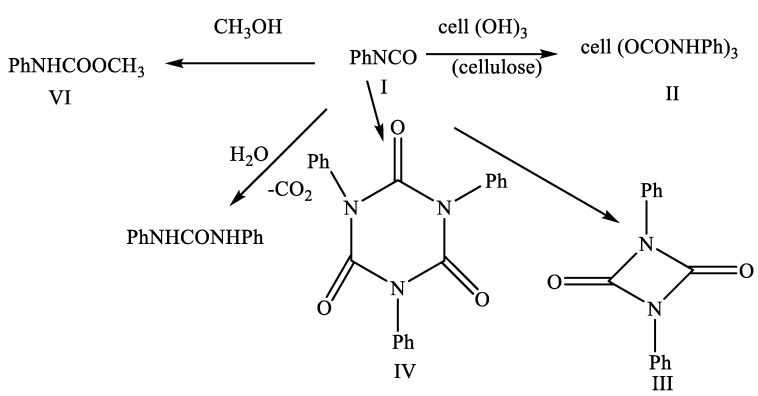
Reactions of phenylisocyanate with cellulose. (Copyright and permission, Wiley—2022).

Bacterial cellulose (BC) carbanilates have DS values ranging from 0.29 to 3.0, dependent on time and molar ratio. In [AMIM][Cl], the cellulose with 3,5-dimethylphenyl isocyanate resulted in the formation of cellulose-tris(3,5 dimethylphenylcarbamate) (CDMPC). [87] Cellulose 6-benzoate- 2,3-phenylcarbamates, a series of Regio selectively-substituted [88] hybrid esters, were synthesized in [AMIM][Cl] using a simple two-step technique [89]. This shows that highly chiral cellulose derivatives with electron-donating substituents on the benzene ring are similar to the commercial Chiral column. Cellulose carbamate is a bio-based, [90] environment friendly substance that be a substitute for petroleum-based polymers. Cellulose carbamate showed a DS of 0.24 in in situ reactive extrusions with urea in the presence of [BMIM][Cl] [91,92]. The effect of carbanilation mixtures containing dimethyl sulfoxide (DMSO) was demonstrated by means of alcohol model substances. The competitive carbanilation was prevented due to steric hindrance of the hydroxyl function. The direct interaction of cellulose and sulfoxide solvent was proven by means of methyl-(2-naphthyl) sulfoxide, which caused the introduction of UV-detectable methylthionaphthyl ether moieties. It is recommended to replace DMSO with solvent components of similar solution behavior, but without the inherent danger of generating oxidants, such as pyridine or DMAc, whenever possible. Cellulose tricarbanilate (CTC) has emerged as a preferred derivative for determining the molecular weight distribution (MWD) of cellulose with the aid of high-performance size exclusion chromatography (HPSEC). Its attributes for this purpose are its stability, and its relative ease of preparation as the fully trisubstituted product. The CTCs (11) for MWD studies are obtained by reacting the cellulose samples with phenylisocyanate (I) in pyridine as shown in Scheme 1 [93].

Cellulose nanocrystal (CNC)-grafted smart polymers have potential applications as adsorbents by providing a simple regeneration process. Carbon dioxide (CO_2_) and temperature-responsive free block copolymers of N-isopropylacrylamide and (2-dimethylaminoethyl) methacrylate with different block lengths were synthesized using reversible addition-fragmentation chain transfer (RAFT) polymerization. In addition, CNC was modified with the similar block copolymers by a surface-initiated RAFT method. The synthesized block copolymers were used in nitrate ion removal from aqueous solutions. Turbidity analysis showed different critical solution temperatures (CST) for the block copolymers with various block lengths. Two different micellar and vesicular morphologies were observed for the self-assembled copolymers at temperatures above and below the CST of the copolymers, which is related to the hydrophobic/hydrophilic block ratio. The presence of CNC, morphology of the self-assembled copolymers, and temperature and time of CO_2_ purging affect the nitrite ion adsorption capacity. The maximum adsorption capacity (420 mg/g) is related to the sample with vesicular assemblies in the aqueous solution and a higher PDMAEMA block length at temperatures below the CST of the block copolymer. N-2 purging resulted in deprotonation of the PDMAEMA blocks and regeneration of the samples. Finally, the CNC substrate can be used in the regeneration of the samples with a simple filtration process, in addition to the stimuli-regeneration process.

### 2.10. Cellulose Toxicity

Cellulose insulation (CI) is a type of thermal insulation produced primarily from recycled material. The recycled material is shredded, milled, and treated with fire-retardant chemicals. The blowing process for installing CI generates a significant quantity of airborne material that presents a potential inhalation hazard to workers. CI was selected for study based upon the high production volume, the potential for widespread human exposure, and a lack of toxicity data; insufficient information was available to determine whether inhalation studies in laboratory animals were technically feasible or necessary. Studies were conducted to characterize the chemical and physical properties of CI aerosols, to evaluate the potential acute pulmonary toxicity of CI, and to assess occupational exposure of CI installers. All samples of the bulk CI were found to contain primarily amorphous cellulose (60% to 65%), with a smaller crystalline component (35% to 40%). The crystalline phase was primarily native cellulose (75% to 85%), with a minor amount of cellulose nitrate (15% to 25%)

### 2.11. Cellulose Modification Toxicity with Small Functional Groups

Respirable cellulose fibers were less toxic in vitro than the mineral fibers, crocidolite and MMVF10. Short-term inhalation of cellulose caused an inflammatory lung response, which resolved, despite continued exposure. The intraperitoneal injection of cellulose fibers induced sarcomas rather than mesotheliomas at the highest dose, whereas the two middle doses each produced a mesothelioma.

In the in vivo tests, cellulose fibers produced harmful effects, including tumors. The tumors in the peritoneal cavity included two rats with mesothelioma, but mainly comprised sarcomas, which are not normally seen with mineral fibers. Long-term inhalation studies with cellulose fibers are recommended. it is expected that the nanometric size of nanocellulose will increase its toxicity compared to that of bulk cellulose. Several toxicological studies have been performed, in vitro or in vivo, with the aim of predicting the health effects caused by exposure to nanocellulose. Ultimately, their goal is to reduce the risk to humans associated with unintentional environmental or occupational exposure, and the design of safe nanocellulose materials to be used, e.g., as carriers for drug delivery or other biomedical applications, such as in wound dressing materials. The available literature has been reviewed for studies in laboratory animals using purified cellulose, as the production of purified cellulose may result in trace organochlorine contamination.

**Figure 5:**
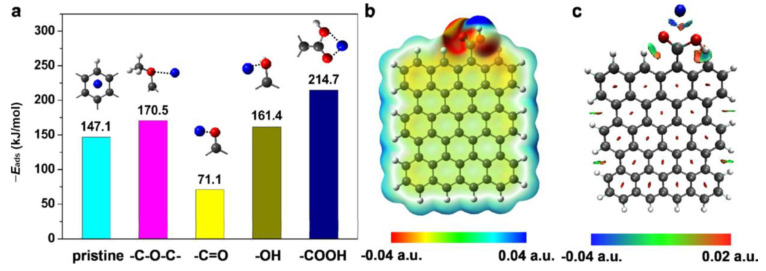
(**a**) Adsorption energy of Na on the pristine carbon surface and near ether, ketone, hydroxyl, as well as carboxyl. (**b**) Electrostatic potential mappings of the carbon surface modified with carboxyl; (**c**) BLYP/DEF2-SVP gradient iso surfaces with *s* = 0.5 au for the adsorption complexes (Copyright and permission, ACS Sustainable Chem. Eng—2022).

Unmodified cellulose has a low level of toxicity [94]. The modification of tiny molecules will modify the surface characteristics and the toxicity. In general, surface modification with neutral chemicals has not resulted in any toxicity issues [95]. The cytotoxicity appears to be slightly increased when cellulose is coated with lignin, as shown in Figure 5 [96,97]. It contains a lot of phenolic groups because they are the initial site of interaction that is inhaled. The two cell lines that were chosen had no influence on the membrane integrity activities. It was discovered that the type of scaffold had a greater impact on the outcome of cellulose [98]. Cyclodextrin is another neutral conjugate [99] with a lot of OH groups [100]. It is commonly used to increase drug solubility [101,102]. Oxygen-containing groups in carbon materials have been demonstrated to be effective in the anodic sodium-ion storage process; however, the effect of specific oxygen-containing groups on the sodium-ion storage in the carbon framework remains to be explored. A selectively modified cellulose-derived hard carbon (BHC-CO2) with a high oxygen content of 19.33 at. % And carboxyl-dominant groups was prepared. The fabricated BHC-CO2 anode exhibits excellent electrochemical performance with a high reversible capacity of 293.5 mA h at a current density of 0.05 A g–1, two times as high as that of the oxygen-deficient BHC-CO2-H2 anode, demonstrating the significant role of oxygen-containing groups in enhancing the Na+ storage. Moreover, the BHC-CO2 anode has excellent high-rate cycling stability with a specific capacity The role of carboxyl on Na+ storage by carbonaceous materials provides theoretical guidance for the oxygen functional group modification of carbon materials to enhance the sodium-ion storage as shown in Figure 5 [103].

The substance plays a potential host role, and its presence on the surface of cellulose had no effect on mouse monocyte cell (J774A.1) and human breast adenocarcinoma cell (MCF-7) proliferation. In addition, the substance had no effect on the intracellular inflammatory response. This type of material is used as human monocyte cell line to test the (J774A.1). The substance is nonimmunogenic, according to the formation of reactive oxygen species. The attaching of amino-containing carbon resulted in a photoluminescent hybrid material to the surfaces of TEMPO-oxidized cellulose when monocyte/macrophage-like cells (RAW 264.7) with more than 500 mg/mL^−1^ concentrations were employed [104]. Surprisingly, the TEMPO-oxidized cellulose utilized looked to be slightly more hazardous than the modified cellulose [105].

In contrast to neutral surfaces, such amino groups would produce potentially harmful cationic charges, and could be thought to hinder cell proliferation. Conjugation with fluorescent dyes increased hydrophobicity, causing aggregation, and as a result, a shift in biological behavior. There was no evidence of cytotoxic behavior [106]. The surface alteration may not have a significant impact on toxicity [107]. The small number of trials conducted were also verified using in vivo models. The toxicity of different celluloses was investigated using an embryonic zebrafish model [108,109].

Cellulose is being developed with the purpose of its use in many applications, in pure and composite forms, from consumer products to pharmaceutics and healthcare products. Respirable cellulose fibers were less toxic in vitro than the mineral fibers, e.g., crocidolite, and artificially made fibers, e.g., MMVF10. Short-term cellulose inhalation caused an inflammatory lung response which resolved despite continued exposure.

## 3. Grafting

Grafting polymerization is a typical method for cellulose modification that involves covalently bonding to generate a branched copolymer. This method gives native cellulose new qualities without altering its inherent features [110,111]. Other techniques, such as ring-opening polymerization (ROP) and reversible addition-fragmentation chain transfer (RAFT) polymerization, can be used to make cellulose graft copolymers [112].

Cellulose grafting reagents are excellent solvents. Its copolymers, with various side chains, e.g., poly(lactide) (PLA), poly(caprolactone) (PCL), poly(dioxanone) (PDO), and polyacrylic acid (PAA), synthesize a variety of amphiphilic cellulose graft copolymer techniques [113]. In BMIMCl medium, the copolymer reactions were able to self-assemble into core-shell micelles. These are employed for hydrophobic carriers in pharmaceuticals, drug delivery, and cell imaging applications. They were used for cationic amphiphilic cellulose copolymers in [BMIM][Cl]. The resulting products had a DS of up to 1.79, indicating that the ROP grafting effectiveness was high. This type of activator regenerated via the electron transfer method to make cellulose-graft-PMMA copolymers [114].

AMIMCl/DMF with 2-bromoisobutyryl groups were initially added to the cellulose backbone as a solvent. Copolymerizing MMA with the resulting macroinitiators yielded the final copolymer products [115]. The copolymers had similar transparency to linear PMMA films, and significantly increased tensile toughness. In BMIMCl media, cellulose created macro-chain RAFT agents. These cellulose-CTAs could then be grafted with PDEAAm and PNIPAM. However, there are few reports of cellulose polymerization by RAFT [116].

## 4. Process and Product Toxicity

A green process should not be harmful to humans or the environment. [117] There is a potential to turn cellulose into a variety of useful advanced materials with less toxicity [118,119]. Cellulose and its derivatives, such as cellulose acetate, carboxymethylated cellulose, and so on, are not a major worry [120]. In coatings and in medicinal applications, these products are widely used [121,122]. They are non-toxic to our environment [123]. As a result, there is a need to increase the anti-solvent properties, decreasing toxicity and improving biodegradability [124,125]. This employed toxicity process is the most significant problem [126].

If they must be used for humans, such fluids are frequently referred to as “green” and safe. There are no proper methods for treating cellulose to maintain its molecular structure. Such properties makes solvents useful in this field because they truly dissolve cellulose rather than degrade it [127].

The original ionic liquid (IL) for dissolving cellulose, [C4mim]Cl, is fairly poisonous. [C4mim]Cl has a median fatal dose (LD50), and has been found to be poisonous to mice at maternally toxic doses [128]. There are an unlimited number of ion combinations that can result in salts. Finding a better cellulose solvent requires not only non-toxic ions, but also a combination of these ions that can dissolve cellulose efficiently. The fundamental mechanism of cellulose dissolving was determined to be the anion’s basicity, [129] which destroys the hydrogen bonds in cellulose. The potentiality of cellulose solvents discovered that the [C2mim]OAc had some promising qualities because the acetate salt has a lower melting point, lower toxicity, and higher cellulose dissolution ability [130]. The biodegradability was clearly enhanced. Additional ionic liquids (ILs) and anti-solvents are required to coagulate the cellulose and recover the ILs [131]. The options with water and ethanol medium are more plentiful. The anti-solvent must form stronger hydrogen bonds than cellulose in order to solvate both ILs. Water is environment friendly, economical, and with no risk, whereas on the other side, the toxicities of organic chemicals studied ranged in many categories. The least toxic chemicals, such as ethanol, methanol, acetone, and acetonitrile, were commonly observed for the regeneration of cellulose from solutions [132].

Another factor to consider for choosing an appropriate anti-solvent solution is saving energy. This green chemistry principle implies that the amount of energy required for any process should be kept to a minimum, and that designing chemical reactions that are low on energy is very desirable. The most common way to process cellulose is to heat it gently, but the exact time and temperature for the best results have yet to be found. Researchers looked at greater temperatures, but for considerably shorter periods. It was noticed that under sonication, a highly concentrated cellulose solution can be created in a short time, whereas others attempted to reduce the temperature for dissolution. It was noticed that cellulose dissolved in phosphonate-based solutions, such as [C2mim][(MeO)HPO_2_, at normal temperature [133,134]. However, it is possible that the amount of energy required and the antisolvent recycling stage will ultimately determine the economic viability of the biomass process. Obviously, the water used to dissolve cellulose may be extracted from its high purity. This can be accomplished with minimal degrading.

Current methods for removing cellulose require lengthy washing procedures and energy-intensive evaporative steps to separate the water [135]. The energy demands of such steps must be decreased, which has sparked a slew of research. Solvent combinations that change form in response to a chemical or physical input are now being explored as viable options. The cellulose feedstock is certainly renewable, as is water when employed as an anti-solvent. Although some research has been published on generating renewable chemicals, this is an intriguing study issue that is still being investigated [136,137].

Quantitative catalytic conversions of wood and cellulosic solids to liquid and gaseous products in a single-stage reactor with a little amount of char are formed. The reaction medium is supercritical methanol (sc-MeOH), and the catalyst, a copper-doped porous metal oxide, is composed of earth-abundant materials. Therefore, in principle, these are suitable for applications as liquid fuels, as shown in Table 1 [138].

The yield of cellulose material is even better than achieved from Kraft pulping techniques. After evaporation of the reconstitution solvent, polyoxometalates can be recovered with [C2mim]OAc [139]. The development of selective catalysts is a very fascinating path for future research. The current ILs for biomass treatment are not biodegradable. Many ILs are recyclable due to their excellent stability. Of the ILs used in cellulose processing, 99.5% recovered with high purity.

Using CO_2_ headspace tests, ILs have been found to be readily biodegradable, with more research underway. Biodegradable ILs will be developed for dissolving cellulose in order to be helpful in various applications [140]. To avoid undesirable byproducts, it is vital to watch for reactions and intervene quickly, [141,142] including the ability to take rapid action to prevent the development of byproducts and the avoidance of sample pretreatment.

## 5. Design for Degradation

Cellulose acetate (CA), a natural polymer, has been modified to have a wide range of characteristics. It gives a different variety of the degree of substitution (DS) because of its strong solubility in common solvents and their molecular weights [143,144]. The most frequent level is a DS of 2.5. CAs can be used in a wide range of industrial products, such as textiles, and plastics that can be thrown away [145,146]. The global output of CA materials is measured in metric tons per year. Many items end up as litter on the ground in composting facilities. It is crucial to know what happens to abandoned CA-based products. This increases the awareness of degradation pathways, which could aid in determining the environmental impact [147]. Due to analyzing exclusively cellulose-degrading organisms such as fungus, the polymer is not biodegradable [148,149].

This type of acetyl esterase enzymes is common in bacteria. The importance of the deacetylation phase became clear. Within the scientific world, CA is now widely accepted as a biodegradable polymer. Photodegradation is another prevalent mechanism for many polymers, lighting the biodegradation mechanism [150,151]. Although the photodegradation of the CA polymer under sunshine is restricted, many consumer items contain additives that allow for improved photodegradation [152,153].

Titanium dioxide is a photooxidation catalyst that causes degradation in sunlight, and is widely used to improve the whiteness of CA materials [154,155]. The combining bio- and photodegradation results in pitting, and increases the material’s surface area for biodegradation [156]. Environmental factors have a significant impact on the pace of the degradation of a substance [157]. Many concepts for developing industrial products that can be built to optimize the degrading environment have been pointed out by researchers [158]. This diversity of concepts for improving degradation rates can be seen in significant patent disclosures [159,160].

The conversion of biomass into biofuels can reduce the strategic vulnerability of petroleum-based systems and, at the same time, have a positive effect on global climate issues. Lignocellulose is the cheapest and most abundant source of biomass, and, consequently, has been widely considered a source of liquid fuel. Cellulosic biofuels are still far from commercial realization, one of the major bottlenecks being the hydrolysis of cellulose into simpler sugars, inspired by the structural and functional modularity of cellulases used by many organisms for the breakdown of cellulose.

Fruit peels, which are usually discarded as agricultural wastes, were utilized to isolate cellulose. The varied amount of isolated cellulose was used as sustainable support with hydrothermally synthesized molybdenum sulphide (MoS_2_) nano-petals via an in-situ approach. In order to evaluate the performance of the catalyst, the photodegradation rate was calculated for RhB dye, as well as industrial effluent, in visible light. The up-gradation in photocatalytic competence was found significant by cellulose-supported MoS_2_ nanostructures as compared to bare MoS_2_ nano-petals due to the slow recombination of electron–hole pairs. The maximum rate was pronounced by employing the cellulose at an amount similar to 500 mg as a support due to the existence of an optimal point where the delay in charge recombination reaches the maximum.

Some researchers found that CA with a degree of substitution (DS) greater than 1.5 could not be degraded by natural organisms, whereas others found that CA with a DS of 2.5 had limited value, owing to deterioration. The main mechanism for degradation is an initial deacetylation process involving chemical hydrolysis and acetyl esterases, which allows the degradation of the cellulose backbone [161,162]. The analysis shows that moisture in the soil caused cellulose acetate fibers to decay significantly, and after a few months, they were completely gone. At the end of the trial, the synthetic textile fibers exhibited no significant modifications [163,164].

The conducting wood-based yarns are produced by a roll-to-roll coating process with ink-based biocompatible polymer. They developed textile yarns which showed a record-high bulk conductivity of 36 Scm^−1^. This was also further increased to 181 Scm^−1^ by adding silver nanowires. The electrochemical functionality of the yarn through incorporation into organic electrochemical transistors was used as a household sewing machine [165]. The aerobic biodegradation of radio-labelled CA, in which they observed the evolution of CO_2_ from in vitro samples with 14C-labeled acetyl carbons, was one of the more compelling degradation investigations. CA showed degrees of substitution of 1.85, 2.07, and 2.57, and it was discovered that higher amounts of acetyl slowed, but did not stop, biodegradation [166]. This was also observed in aerobic test methods for degrading CA films, which had in vitro enrichment cultivation methodology and a wastewater treatment system with activated sludge [167]. CA films were degraded in 2–3 weeks by the enrichment culture, as evidenced by a 67 percent weight loss, respectively. The wastewater treatment of the industrial system also produced the same degradation, albeit at a slower rate, with major alterations in the films taking 10 weeks. The researchers made it clear that such degrading processes only increased the concentration in the natural microbes’ abilities [168]. The biodegradation capability was preserved, but the rate of breakdown was altered. Anaerobic environments have also been proven to degrade CA. The DS was increased (0.82–2.4) by incubating them for 98 days with a specific culture. It was noticed that the CA of DS 1.25% were considerably damaged.

The biodegradability of CA films had DS values ranging from 1.7 to 2.5. At a temperature of 53 °C, the materials were subjected to biologically active aerobic test vessels [169]. It was noticed that after 7 and 18 days of incubation, the films had totally vanished. The pitting of the films by bacteria, as well as the modest changes in DS and molecular weights as the polymer decomposed, were noteworthy observations [170]. The random breakage of the polymer was thought to be a result of low molecular weight. The products would disperse away from the bulk components and quickly be digested by microorganisms. The anaerobic conditions in a bioreactor with cellophane and a CA film showed DS = 1.7. The CA film entirely deteriorated after one month. Both aerobic and anaerobic bacteria produce the entire range of hydrolases [171], including esterases. They destroy naturally occurring acetylated polysaccharides, such as acetyl-4-O-methyl glucuronoxylan, acetyl galacto glucomannan, and chitin [172]. Fungi are key players in biotechnological applications. Although several studies focusing on fungal diversity and genetics have been performed, many details of fungal biology remain unknown, including how cellulolytic enzymes are modulated within these organisms to allow changes in main plant cell wall compounds, cellulose, and hemicellulose, and subsequent biomass conversion. With the advent and consolidation of DNA/RNA sequencing technology, different types of information can be generated at the genomic, structural, and functional levels, including the gene expression profiles and regulatory mechanisms of these organisms during degradation-induced conditions. This increase in data generation made rapid computational development necessary to deal with the large amounts of data generated. The origination of bioinformatics, a hybrid science integrating biological data with various techniques for information storage, distribution, and analysis, was a fundamental step toward the current state-of-the-art in the postgenomic era. The possibility of integrating biological big data has facilitated exciting discoveries, including identifying novel mechanisms and more efficient enzymes, increasing yields, reducing costs, and expanding opportunities in the bioprocess field.

Fei et al. found that the time required for CA breakdown in laboratory composting conditions was influenced by changes in the compost mixture composition, particularly the wet content. The DS of the CA material after 35–50 wt% loss was evaluated by researchers. They discovered that the DS of the residual material did not change significantly [173]. They deduced from this that the degradation was entirely biological, and not caused by chemical deacetylation [174,175]. Microbial cellulase is under intensive investigation due to its expected use as a tool for the biological degradation of cellulosic wastes. The production of cellulase by *Trichoderma viride* in 5% wheat bran medium with glucose as the carbon source was studied. The biological degradation of cellulosic wastes, such as banana stem, waste newspaper, waste plane paper, etc., has been observed during the growth of the organism due to the production of the cellulase activity. The results indicated that the organism produced two cellulases, one in the earlier phase, and the second in the later phase. The cellulase produced in the earlier phase was in a significant quantity, and was subject to induction by the cellulosic substrates included in the medium. The banana stems, particularly in the shredded form, underwent a large degradation, as this material, after inclusion in the medium, underwent a 90% loss in weight. The results help in the production of sugar and ethanol from no-cost solid wastes, and will offer a partial solution to the ongoing food and energy crises, along with the effective disposal of solid waste to keep the environment clean. Microbial depolymerization of plant cell walls contributes to global carbon balance, and is a critical component of renewable energy. The genomes of lignocellulose-degrading microorganisms encode diverse classes of carbohydrate-modifying enzymes; although, currently, there is a paucity of knowledge on the role of these proteins in vivo. A comprehensive analysis of the cellulose degradation system in the saprophytic bacterium Cellvibrio japonicus was performed. Gene expression profiling of C. japonicus demonstrated that three of the 12 predicted, 4 endoglucanases (cel5A, cel5B, and cel45A) and the sole predicted cellobiohydrolase (cel6A) showed elevated expression during growth on cellulose. Targeted gene disruptions of all 13 predicted cellulase genes showed that only cel5B and cel6A were required for the optimal growth of cellulose. The analysis also identified three additional genes required for cellulose degradation: lpmo10B encodes a lytic polysaccharide monooxygenase (LPMO), whereas cbp2D and cbp2E encode proteins containing carbohydrate-binding modules and predict cytochrome domains for electron transfer. CjLPMO10B-oxidized cellulose and Cbp2D demonstrated spectral properties consistent with redox function. Collectively, this report provides insight into the biological role of LPMOs and redox proteins in cellulose utilization, and suggests that C. Japonicus utilizes a combination of hydrolytic and oxidative cleavage mechanisms to degrade cellulose.

## 6. Applications of Cellulose

Cellulose is a linear polymer of anhydrous units linked by one to four carbon atoms by a β-glycoside bond, possessing microscopic properties which have important effects on their microscopic energy, such as rheology, colloidal stability, etc. Organic–inorganic hybrids have attracted great attention over the past decades for their combined properties of both organic and inorganic components. Cellulosic materials are well studied for renewable purposes if the degree of substitution with functional groups is low, because they are biodegradable. Due to the presence of an activated hydroxyl group, cellulose and its derivates have the advantage that they can easily modify with other related functional groups, such as oxidation, esterification, etherification, grafting copolymerization, cross-linking reactions, etc.

The application of cellulose has gained increasing attention in recent years, driven by the desire for sustainable products. Cellulose can replace petroleum-based plastics because it can be converted to biodegradable and nontoxic polymers from sustainable natural resources. These products have increasingly been used as coatings, self-standing films, and paperboards in food packaging, owing to their promising mechanical and barrier properties. With the presence of a large number of functionalities within pristine cellulose and its derivatives, these building blocks provide a unique platform for chemical modification via covalent functionalization to introduce stable and permanent functionalities to cellulose. A primary aim of chemical attachment is to reduce the probability of component leaching in wet and softened conditions, and to improve the aqueous, oil, water vapor, and oxygen barriers, thereby extending its specific use in the food packaging field. However, chemical modification may affect the desirable mechanical, thermal stabilities, and biodegradability exhibited by pristine cellulose.

### 6.1. Metal Adsorption

**Figure 6:**
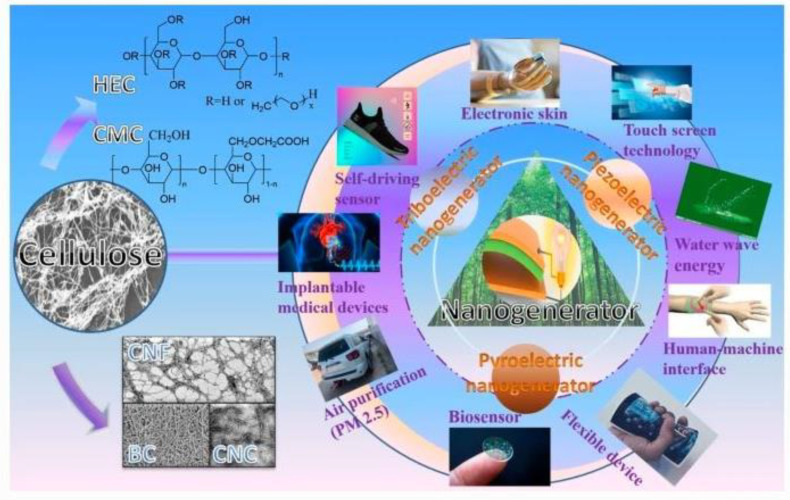
Recent strategies and advances in cellulose and its applications were comprehensively reviewed.

Cellulose-based nanogenerators include nanocellulose, cellulose derivatives, and cellulose-based composites. Strategies and principles of improving the output performance of cellulose have been demonstrated, instead of traditional petroleum-based polymers as the substrate material, and can effectively solve the environmental pollution problem caused by the non-biodegradability of conventional polymers, as shown in Figure 6 [176]. The heavy metals’ recycling and their detection in water is a fascinating topic that is garnering more attention [177]. It is significant from an environmental standpoint, and the recovery of metals from wastewater is also of interest to the industry [178].

Gold nanoparticles (AuNPs) modified by cellulose ester membrane and laser-induced ionization mass spectrometry were used to detect arsenic (LDI-MS). When AuNPs reacted with Pb(II) ions in alkaline solutions [179], Au-Pb complexes PbO and Pb(OH) developed on the AuNP surface quickly. Lead shells formed on the AuNPs surfaces reacting with additional AsO_2_ As a result, there was a large visible aggregation of AuNPs in the solution. These Pb(II)AuNPs was capable of detecting AsO_2_ at low concentrations, such as 0.6 M, with good selectivity. Such an approach might be used to detect AsO_2_- in water samples [180]. Gurung et al. studied precious metal adsorption. They concentrated on the absorption and preconcentration of gold from wastewater to make an adsorbent for metal nanoparticles [181,182]. They used the cross-linking method for dried cellulose with epichlorohydrin (ECH) to change the surface with N-aminoguanidine (AG) functional groups. The dried cellulose did not adsorb on the metallic species [183].

The raising concentration of competing ions had no influence on metal adsorption. The base-metal ion adsorption was almost undetectable [184]. An anion exchange method was thought to be the best adsorption mechanism by electrostatic contact. The base metals exist in hydrochloric acid (HCl) media as cationic species. The degradation of the cellulose crystalline results in a porous material with improved adsorption affinity. This was thought to be the cause of the sulfuric acid (H_2_SO_4_) cross-linked materials. It helps to increase the adsorption effectiveness. Comparing with Pd(II) and Pt(IV), the H_2_SO_4_ cross-linked adsorbent had a substantially better adsorption capacity towards Au(III). It is a viable material for the recovery of gold (Au) in wastewater [185].

Firstly, it was converted into elemental gold after the adsorption of Au(III). This increased the reduction potential of Au(III) relative to Pt(IV) and Pd(II). The Sulf-AG-cellulose adsorbent used to separate trace quantities of the metals from industrial effluents was also concluded from these column trials. This conjugated bovine serum albumin (BSA)-AuNCs with modified cellulose is used for the detection of Hg^2+^ in fish and tap water samples. The cellulose paper was activated in a sodium hydroxide solution before being esterified with the pyridoxal conjugated BSA-AuNCs-free carboxylic acid group. A strip tested for Hg^2+^ concentrations ranging from 1 mM to 1 nM. A significant color change from red to blue was seen, suggesting that mercury ions [186,187] had been detected [188,189].

### 6.2. Surface-Enhanced Raman Scattering

Low detection limits and a potential technology for detecting environmental toxins were also used i.e., surface-enhanced Raman scattering (SERS). It differentiated distinct molecular orientations of adsorbed molecules, allowing for single-molecule detection [190,191]. This method did not result in a uniform distribution of AuNPs on paper. Upon starting, agglomerates developed. The nitroaniline (NA) adsorption was shown to be distinct P-NA molecules [192]. There was a flat layer of gold coated on the filter paper. In colloidal conditions, they stood perpendicularly on their nitro group surfaces. The Au adsorption density on filter paper had a significant impact on the P-NA SERS spectrum. Bifunctional dip-catalysts based on AuNPs on filter paper were created by dipping filter paper in a concentrated AuNPs solution. There were strong van der Waals contacts and hydrophobic effects at the metal cellulose interface. The spontaneous self-assembly of oleylamine-stabilized AuNPs resulted in the formation of a robust catalyst. The reduction was complete in half an hour. The catalytic efficiency was demonstrated for a period of twenty reaction cycles. SERS showed high AuNPs-loaded substrates. Such extensive surface coupling occurred between the densely packed AuNPs on the paper substrate. The peak contributions to the distinctive functional groups in the SERS spectra changed with time [193,194].

The spatial deformation of the Au-cellulose hydrogel density of SERS increased. By reducing AuNPs in situ in the presence of bacterial cellulose (BC) fibers, AuNP-bacterial cellulose hydrogels were created. The SERS signal was significantly enhanced due to the spatial deformation of the hydrogels drying process [195,196]. A microstructure of uniform multilayers cellulose compounds characterized the hydrogel. The distance between those layers reduced throughout the drying process, and the AuNPs on the bacterial cellulose nanofibers created a number of hot spots. A SERS signal increase was measured for 4-fluorobenzenethiol and phenylacetic acid by contracting the hydrogel and bringing the molecules close to AuNPs. The influence of drying on signal amplification and the production of more hot spots were produced as a result. The reduction of tetra chloroauric acid in the presence of bacterial cellulose (BC) and the use of trisodium citrate resulted in a significant amplification of the SERS signal as an in situ reducing agent [197]. The pH of the solution was able to reverse this connection without harming the SERS substrate. The colloidal Au was synthesized by a redox method [198].

It was found that Au aggregation had a significant impact on the SERS effect of C60/C70. Because the influence of solvents in the C60/C70 solution and hydrosols could be avoided, the dried, coated gold showed a high SERS activity [199,200]. The SERS subtract intensity of most peaks of colloidal Au coatings increased and then slowly decreased depending on the saturation [201,202].

### 6.3. Antioxidant

The AuNP production of unbleached kraft softwood fibers reducing/stabilizing also served as a substrate [203] for AuNP immobilization. Here, the OH groups of cellulose can convert a small amount of tetrachloroauric acid. The reduction of lignin was required for Au(III) to Au(0). The solution of 2,2- diphenyl-1-picryl-hydrazyl (DPPH) in both conditions was used to assess the antioxidant activity. In both conditions of light, the composite had a significantly increased DPPH radical scavenging capability. Many pores between the fibers were discovered in the unbleached kraft paper-AuNPs composite sheets [204]. As a result, DPPH molecules’ adsorption affinity was enhanced. This adsorption resulted in enhancing the antioxidant performance [205,206].

### 6.4. Bio-Sensors

Bienzyme biosensor-immobilized GOx (glucose oxidase) and horseradish peroxidase (HRP) in the gold-BC nanocomposite were investigated. We utilized the composite ampere to metrically quantify glucose in human blood samples. In Au-BC nanocomposites, both biocatalytic activity were highly retained. The bienzyme biosensor was stable and showed a fast reaction rate, a low detection limit (2.3 M), good anti-interference, and repeatability [207].

For enzyme immobilization and biosensor fabrication, Au-BC nanocomposite was employed. Heme proteins were immobilized on the surface, such as hemoglobin (Hb), myoglobin (Mb), and horseradish peroxidase [208,209]. The biosensor’s sensitivity increased as the number of nanoparticles increased. AuNPs functioned as a link between HRP and BC. The biosensor’s sensitivity was greatly improved because of AuNPs’ strong electrical conductivity and electron transfer acceleration capabilities. The range of concentration was 0.3 M to 1.00 mM. The HRP-based biosensor demonstrated a good linear response, with a detection limit of 0.1 M. Due to its higher performance, the Au-BC biosensor was shown to be a superior immobilization matrix [210,211].

Au-BC bio-composite using an H_2_O_2_ biosensor employed HRP as a model enzyme, using hydrogen peroxide for activation. The oxidation of substrates accelerates HRP with iron heme prosthetic groups. H_2_O_2_ is widely employed as a bioactivity test for HRP. To detect hydrogen peroxide, hydroquinone (HQ), an electron mediator, was used in peroxidase-based ampere metric biosensors [212]. The AuNPs function as tiny conduction centers. It allows the HQ and the bulk electrode surface, leading to high H_2_O_2_ responsiveness. It was also discovered that including AuNPs in the BC network structure for the biosensor’s catalytic activity substantially determined the H_2_O_2_. Most other HRP/AuNPs-based matrices show better performance, such as AuNPs-CaCO_3_/silica sol-gel (1M), AuNPs/sol-gel (2M), etc. The Au-BC bio-composite could be used to immobilize an enzyme. Au-BC hybrid nanofibers were used on the surface of an electrode to create a biosensing platform by adding laccase and Nafion. For electrocatalysis dopamine, the sensor was evaluated with low detection limits and a wide linear range, respectively [213,214].

The sensor shows good stability and was successfully employed for the detection of hydroquinone in water samples. They also identified DNA-cross-linked AuNPs on both hydrophilic and hydrophobic paper. The aggregated gold dissolved into AuNPs by DNase-I and adenosine, resulting in a bright red hue on paper in a very short time. The gold nanoparticles’ color change was generated by the surface plasmon resonance [215]. For this biosensing experiment, both hydrophobic and PVA-coated hydrophilic paper were found. It was suitable for surface drying, and caused “bleeding” with the early termination test on uncoated hydrophilic paper. The biosensing ability was maintained at temperatures up to 90 °C, and were thermally stable. Furthermore, afterwards, AuNP aggregate coated paper was stored for many weeks. The detection limit of DNase-I was found to be in the low nM range. The AuNP aggregate-coated paper could be used for disease diagnostics and water quality. They could be used for biosensor in thiols by exposing mesoporous photonic cellulose to 2-mercaptoethanol (2-ME). After being exposed to 2-ME, color changes were observed, indicating that the host matrices, i.e., microporosity, allow analytes to reach the NPs, potentially making the composite suitable for a sensor. The hue shift became more extreme as the ME concentration increased [216].

A glucose biosensor used Au nanohybrids and PDAA-CNCs as a helper. Imaging and free-standing fluorescence sensors are all possible with the photonic AuNC-CNC films. Such films were able for the spontaneous emission of Au-CNCs. This could be used for the photonic crystal photoemission coupling phenomena. The nematic chiral structure allows for fluorescence emission amplification. The AuNPs were stabilized by BSA, which includes amino groups, and their surfaces were enriched with electrons. Amine-functionalized fluorescence donors exhibit an optical response after being exposed to electron-deficient aromatic rings [217].

Using 2,4,6-trinitrophenol (TNP) as a model, the AuNC-CNC film was evaluated for nitroaromatic explosive sensing. The composite film was validated to be a portable visual TNP sensor. Scanning electronic microscope (SEM) pictures indicated the AuNC-chiral CNCs’ nematic ordering exposure to a TNP ethanol solution film that remained unbroken. The intensity of the AuNC-CNCs sensors fluorescence decreased as the TNP concentration increased. In recovery tests, the sensor’s performance and stability were established. The ionic liquid, 1-butyl-3-methylimidazolium hexafluorophosphate, was used to make an electrochemical oxygen sensor, which exhibited high sensitivity for O_2_. This material has the potential to be low-cost and ecologically friendly. Cysteine is used in biological samples using carboxymethyl cellulose-functionalized gold nanoparticles (CMC-AuNPs) [218,219].

It was found that it has a strong colorimetric selectivity. When added to NaCl solutions, the aggregation started, which causes color changes in the UV-Vis absorption spectra [220]. CMC was assumed to move away from the AuNPs’ surface because cysteine binds to it via the Au-S connection. The introduction of NaCl improves the selectivity and increases particle aggregation [221], indicating that the sensor might be employed as a cysteine-specific colorimetric visual probe. The range of the CMC-functionalized AuNPs was detected from 10.0–100.0 M [222,223].

### 6.5. Enzyme Immobilization

Drozd et al. developed a platform for the immobilization of enzymes [224]. They conjugated cyclodextrin glycosyltransferase (CGTase) and alcohol oxidase as an active matrix [225]. To activate, carboxylic acid groups were first inserted. The carbodiimide coupling was used for connecting the enzymes [226]. The specific enzyme activity of CGTase and alcohol oxidase was discovered to be around 70–95%. A similar approach was used to immobilize papain by the same group. After stabilizing magnetite nanoparticles, they used CNC-Fe_3_O_4_NPs-AuNPs as a matrix for the covalent isolation of papain from the solution (Fe_3_O_4_NPs). The AuNPs were used to create a large surface area for enzyme conjugation. The magnetite and AuNPs were dispersed on the surface [227]. Carbodiimide coupling was used for the activated nanocomposite surface [228]. Under normal conditions, a papain loading of 186 mg matrix was attained. It was revealed that the immobilized enzyme retained 95% of its initial activity [229].

### 6.6. Separation of Protein

You et al. coated cellulose with gold Qatarized capillaries (Au-QC), enabling high-resolution protein separation by electrophoresis [230]. In terms of separation, the Au-QC performance had no effect on pH solution. It is closely related to protein separation with similar primary sequences. The structures of AuNP nanocomposites’ creation increased the performance [231]. The Au-QC was also successfully separated in glycoproteins. In addition, the Au-QC detected lysozyme in milk and tear samples, indicating that the Au-QC coating has a potential use in biological analysis [232,233,234], providing a new method for detecting lysozyme from various sources.

### 6.7. Antimicrobial Activity

Johnston et al. studied antibacterial activity of Nanogold unbleached kraft composite fibers against Staphylococcus aureus germs [235]. The fibers were placed on inoculated agar gel with Staphylococcus aureus bacteria, and incubated at 35 °C for one day. Microorganisms grew on the agar gel fibers due to the Au antibacterial characteristics. As a result, it was discovered that effective antibacterial action could be observed even at very low gold concentrations [236,237].

### 6.8. Conductivity

The cellulose in AuNPs’ aqueous dispersions were regenerated by Turyanska et al., and AuNP networks were formed on cellulose sheets. The mechanism of electronic conduction in composite films is totally dependent on its resistivity. At low temperatures and high magnetic fields, the negative magnetoresistance was discovered. AuNPs’ magnetoresistance is proportional to their spin polarization. The researchers tried their best to understand the impact of size and surface properties on AuNP magnetoresistance [238]. The defect-free cellulose matrix has electrical properties. They interconnected the self-supporting cellulose network with AuNPs. The hybrid films exhibited electrical conductivity that was dependent on AuNP loading [239,240]. The same group of AuNPs also developed with a high content by immersing them in colloidal AuNPs solutions, containing high concentrations and a large nanoparticle size. A concentration of 12.7% tunneling stable conductivity was achieved. Hybrid film content of 44% of Au was a substantial electrical coupling between adjacent nanoparticles. Flexible electrochemical electrode substrates have been successfully employed with hybrid films containing AuNPs, i.e., 46.7%. At concentrations below 109 M, Ca cations could detected by using electrodes from hybrid films [241,242]. As a result, conductive hybrid films could be used to make biological sensors and electronic devices [243].

### 6.9. Other Applications

Cellulose membranes with gold-coating are utilized as semiconductors (solar cells) for the water filtration method [242]. Due to this inclusion of cellulose, Hu et al. developed an AuNP-CNC-PVA membrane with improved mechanical strength. Continuous-wave visible laser irradiation with low-intensity was used to achieve photothermal heating. This type of effect was enhanced when the concentration of the nanoparticle was higher. The light scattering wavelengths were also induced by the cellulose, increasing with the total intensity of the photothermal event [243].

SiO_2_-cellulose paper with Au anodes produce a green substrate for flexible top emission organic light-emitting diodes (TE-OLEDS). Compared with the TE-OLED and the use of Ni anodes, the TE-maximum OLEDS’ brightness was determined to be 3516 cd m2, which is around three times brighter. This is much higher than a bacterial cellulose film (0.085 cd A1 at 200 cd m^2^) [244].

The use of chiral nematic structures for fluorescence amplification provides a powerful and long-lasting approach for creating ultrasensitive optical devices. It has a lower detection limit and a better nonlinear response. AuNPs which were produced in situ inside cellulose fibers matrix confirmed that the color of each nanocomposite is linked to the assembly method. The AuNPs’ thickness provides an additional parameter for optical characteristics. These composites showed potentiality for security paper applications.

### 6.10. Advantages and Disadvantages of Cellulose

Alternative renewable energy must emerge to sustainably meet the energy demands of the present and future. The cellulose content of the lignocellulosic feedstock is significant. However, a relatively new area on the use of cellulose is still in its early stages; hence, the advantages of using cellulose as feedstock for industrial and commercial purposes. Furthermore, current green and sustainable methods for cellulose production have a higher yield, which could be attributed to the modified structural and lattice arrangement for its porous volume and polymerization.

The cellulose immobilization rate was strongly affected by conditions such as the carrier concentration and the carbodiimide (EDC) concentration. The results showed that the immobilized enzyme pretreatment could loosen the cell well of the corn stalk, and partly remove lignin and hemicelluloses from the corn stalk, which was beneficial for the further extraction of cellulose by other treatment methods. Cellulose has the characteristics of a porous and large specific surface area. In addition, its surface contains many hydroxyl groups, which is very convenient for modification. The corresponding cellulose derivatives can be obtained by physical, chemical, biological, and other methods, which have a very high development potential and commercial utilization value. With the development of human society, the demand for cellulose as a green and renewable resource will increase day by day, and the utilization of cellulose will continue to become the focus of scientific research.

The percolation network of low-content rigid particles can effectively reinforce biomass polymers, which can solve the problem of renewable materials with low mechanical properties, such as the low strength and Young’s modulus, and bad elongation at break. The advantage of cellulose nanocrystals (CNCs) is the high aspect ratio and a nonuniform modification on their chemical surface structure, where the modification focused on their middle part. The modified part could enhance the compatibility between CNCs and hydrophobic biomass polymers. This type of modification strategy can control the multiple networks in weakly compatible composite systems, and offer EUG-based materials, which usually suffer hard molecular movement, mechanical properties greater than the common petroleum-based rubbers and elastomers.

Man-made cellulosic fibres (MMCFs) were utilized as reinforcement in unidirectionally (UD) manufactured thermoset composites, and were compared to several commercial UD flax fiber products. Specimens were prepared using a vacuum-bag-based resin infusion technique, and the respective laminates were characterized in terms of void fraction and mechanical properties. The MMCF laminates showed better mechanical performance when compared to flax fiber laminates. The failure mechanisms of MMCF laminates were noted to differ from those of flax-reinforced laminates. The results demonstrate the potential of MMCFs as a viable alternative to glass fiber for reinforcement on a larger scale of UD laminates. These results were utilized in the biomaterial demonstration vehicle. In recent years, research and innovation on cellulose-based food packaging have provided solutions to help reduce our dependency on fossil fuels. The advantages of cellulose films are the abundant natural origin of cellulose and its complete biodegradability.

However, the ability of chemical modification to mitigate the hydrophilic nature of cellulose and to ensure that the mechanical and barrier performance of cellulose-based materials is comparable to that of petroleum-based counterparts. Several mechanisms have been used to achieve mechanical and barrier performances that are equivalent to, and even exceed those of, the corresponding petroleum-based materials. Blending cellulosic material with additives, such as plasticizers and active agents, has been demonstrated to improve the mechanical features and prolong the food storage ability of cellulose-based materials without greatly reducing their biodegradability. With respect to industrial-scale applicability, chemically-modified hydrophobic cellulose has been adequately explored to establish its capacities for current and future applications. Given the range and diversity of options available for chemical hydrophobization strategies, there are excellent prospects for extending the application range of cellulose-based materials, comparable to that of fossil fuel-based materials. Without the drawbacks of resource depletion, pollution, and recalcitrance, it has the potential to act as a potent alternative in the market, and could contribute to future prosperity for the planet and its inhabitants.

## 7. Conclusions

Cellulose is the most prevalent renewable material on Earth, and has many interesting features. Turning it into homogenous cellulose derivatives via chemical modification is an effective method. In this review, we tried our best to briefly discuss the recent modification techniques in solvent systems. They have their own set of benefits and drawbacks. Issues remain, such as the high cost, easy way of preparation, and non-degradability. Rapid cellulose dissolving is possible in aqueous alkali systems. The etherification in alkali solutions is the most common method of functionalization. This type of reaction on cellulose modification in alkali solutions is largely limited. The broader scientific community’s input is required. Important challenges, such as the easy preparation, toxicity evaluation, and recovery of solvents, still need to be examined in response to the green chemistry. The synthesis of a large number of chemical reaction steps, i.e., bio-derived solvents generated from renewable sources, will be an important direction. Many bio-based compounds might be considered to be made greener. In the future, the modification of cellulose and application-oriented research will be critical. The market is changing in a dramatic way from traditional petroleum-based development toward bioresource-based development. The green conversion of cellulose and their functional derivatives with improved efficiency and high selectivity should be the focus of future studies. Furthermore, more research into the choice of reaction mediums, reactants, and modification procedures is required.

## Figures and Tables

**Table 1:** Effects of reaction time and substrate loading on cellulose solubilization.

Cellulose (mg)	Time (h)	Remaining Solid (mg)	Conversion (%)
200	0.5	180	60
200	2	95	>99
200	4	102	>99
200	8	101	>99
100	8	99	>99
400	8	99	>99
600	8	100	>99
200	8	98	>99

## Data Availability

Not applicable.
